# Effect of the CAD/CAM Milling Protocol on the Fracture Behavior of Zirconia Monolithic Crowns

**DOI:** 10.3390/ma17122981

**Published:** 2024-06-18

**Authors:** Andressa Restani Oliveira, Natalia Ulmi Ziglioli, Susana M. Salazar Marocho, Julian Satterthwaite, Marcia Borba

**Affiliations:** 1 Graduate Program in Dentistry, University of Passo Fundo, BR285, Passo Fundo 99052-900, RS, Brazil; andressa_restani@hotmail.com (A.R.O.); natliaziglioli@gmail.com (N.U.Z.); 2 Department of Biomedical Materials Science, University of Mississippi, 2500 N. State St., Jackson, MS 39216-4505, USA; ssalazarmarocho@umc.edu; 3 Division of Dentistry, University of Manchester, Coupland Building 3, Manchester M13 9PL, UK; julian.satterthwaite@manchester.ac.uk

**Keywords:** CAD/CAM, ceramics, fixed dental prosthesis, zirconia

## Abstract

Although advancements in CAD/CAM technology allow for more personalized treatments, it is not clear how modifications in the CAD/CAM milling process could affect the restoration surface conditions and their mechanical behavior. The objective of this study was to evaluate the effect of different CAD/CAM milling protocols on the topography and fracture behavior of zirconia monolithic crowns (3Y-PSZ) subjected to a chewing simulation. Monolithic 3Y-PSZ premolar crowns were milled using three protocols (*n* = 13) (slow (S), normal (N), and fast (F)). Crowns were cemented on a dentin analog abutment and subjected to mechanical aging (200 N, 2 Hz, 1,500,000 cycles, 37 °C water). Surviving crowns were subjected to compressive load test and analyzed using fractography. Fracture load data were analyzed with two-parameter Weibull analysis. The surface topography of the crowns was examined with a stereomicroscope and a 3D non-contact profiler. All crowns survived the chewing simulation. Crowns milled using the F protocol had the greatest characteristic fracture load, while crowns produced with the S protocol showed high Weibull modulus. Groups N and S had a more uniform surface and detailed occlusal anatomy than group F. The CAD/CAM milling protocol affected the topography and mechanical behavior of 3Y-PSZ monolithic crowns.

## 1. Introduction

Monolithic polycrystalline zirconia dental prostheses are conventionally produced using computer-aided design/computer-aided manufacturing (CAD/CAM) technology [1,2,3,4]. During CAM machining, partially or fully sintered zirconia blocks or discs are milled using diamond burs to produce the final restoration. The quality of the milling process can be determined by various factors, such as the size, shape, and abrasiveness of the burs; number of axes of the CAM unit; machining speed, time, and path; and, properties of the restorative material [5,6,7,8,9,10,11,12,13]. New functionalities that allow the operator to choose among different milling protocols have been recently added to the CAD software, making it possible to tailor the CAM protocol according to the treatment requirements [14]. Different milling protocols could produce different surface characteristics [7,8], which could also affect the internal fit, marginal adaptation, and ultimately the failure behavior of the restorations [9,10,12,14,15,16,17,18].

Ceramic CAD/CAM blocks and discs are produced under controlled industrial conditions, resulting in homogeneous materials [3,4]. However, the literature shows that, despite the reduction of processing flaws, the CAM subtractive manufacturing process can also introduce surface and sub-surface damage to the ceramic material in the form of micro-cracks [9,12,13]. Once these micro-cracks are under stress and in a humid environment, they can propagate and lead to restoration failure [19]. Fractographic analyses of clinically failed ceramic prostheses have shown that fracture can be originated from surface flaws introduced by processing steps, such as CAD/CAM machining and surface treatments, and also by contact damage associated with chewing and occlusal wear [4,19,20,21].

The composition and microstructure of polycrystalline zirconia have been modified aiming for more translucent materials to produce monolithic restorations [3,4]. One approach was to improve the translucency of 3 mol% yttria partially stabilized zirconia (3Y-PSZ) by sintering the ceramic at higher temperatures and achieving greater density. The content of alumina sintering additive, that could act as light scattering centers, also has been reduced [3,4]. Although these modifications result in only minor translucency improvements, the mechanical properties, such as hardness, fracture strength and toughness of the polycrystalline zirconia were preserved [22,23]. Therefore, 3Y-PSZ can be indicated to produce monolithic dental prostheses in areas of high mechanical demand and mild aesthetic requirements [4].

In vitro mechanical tests are important tools to characterize novel processes and materials in order to support their clinical application. Yet it is important to simulate the conditions in which the restorative materials are subjected clinically [4,19,24]. Clinical variables, such as chewing load and frequency, as well as the presence of humidity and human body temperature, should be included in fatigue tests and chewing simulation protocols [16,19,25,26,27]. Cyclic loading of ceramic materials in a humid environment can lead to slow crack growth and affect the toughening mechanisms of materials, resulting in fatigue failure or degradation of their properties [19,27,28]. Moreover, the structural reliability of ceramics is associated with the size, density, and distribution of the flaws, which can be indirectly assessed using a Weibull analysis [29,30].

Therefore, the objective of this study was to characterize the topography and failure behavior of monolithic zirconia (3Y-PSZ) crowns produced with different CAD/CAM milling protocols (slow, normal, and fast), subjected to a chewing simulation. The study null hypothesis is that the milling protocol does not affect the (1) surface topography, (2) fracture load, and (3) reliability of monolithic crowns.

## 2. Materials and Methods

Monolithic 3 mol% yttria partially stabilized zirconia (3Y-PSZ; VITA YZ HT White, VITA Zahnfabrik, Bad Säckingen, Germany) premolar crowns were manufactured using three different CAD/CAM milling protocols (S—slow, N—normal, and F—fast). Crowns were subjected to chewing simulation and tested under compressive load until fracture. The surface topography of the crowns was qualitatively analyzed with a stereomicroscope and a 3D non-contact profiler. The investigated materials are described in Table 1.

A dentin analog material (glass-fiber reinforced epoxy resin, G10, Jiujiang Xinxing Insulation Material Co., Jiujiang City, China), with similar adhesive and elastic properties to human dentin, was used to produce abutments simulating a simplified preparation for a maxillary second premolar crown [7,16]. The abutments were produced with 6 mm height, 8 mm diameter, total occlusal convergence angle of 12°, and a chamfer finish line (1.2 mm radius between the cervical area and axial wall) [16].

**Figure 1:**
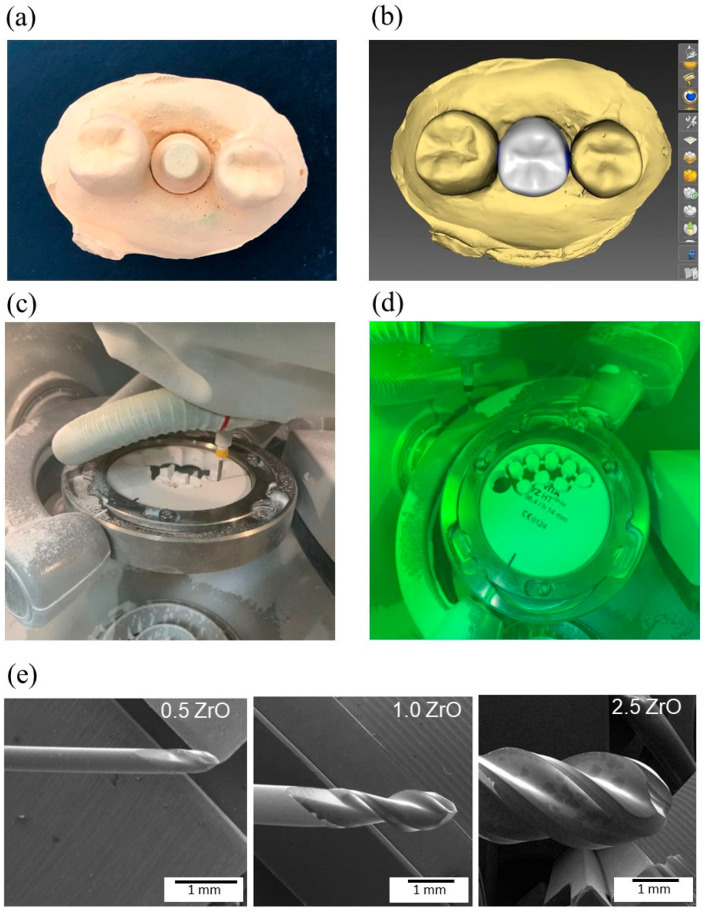
CAD/CAM digital flow. (**a**) Plaster model of the abutment and adjacent teeth. (**b**) CAD design of a maxillary second premolar monolithic crown. (**c**) Dry milling of a partially sintered zirconia disc using the MC X5 CAD/CAM unit. (**d**) Batch of zirconia crowns after milling. (**e**) Representative SEM images of the three types of CAD/CAM milling burs.

The abutment was placed between two adjacent artificial teeth in a master model. The model was duplicated in a special type IV plaster (Figure 1a), which was scanned using an extraoral scanner (InEos X5, Dentsplay Sirona, Bensheim, Germany). The images were imported into inLab CAM 19.0 software (Dentsply Sirona, Bensheim, Germany) and used to design the maxillary second premolar monolithic crown (80 μm cement space) (Figure 1b).

The InLab MC X5 equipment (Dentsplay Sirona, Bensheim, Germany) was used to produce the crowns with three different milling protocols (*n* = 13): (S) slow, (N) normal, and (F) fast (Table 2, Figure 1c,d). The same sequence of diamond-coated burs (Dentsply Sirona, Bensheim, Germany) was used for the three protocols: Bur 0.5 ZrO_2_ DC, Bur 1.0 ZrO_2_ DC, and Bur 2.5 ZrO_2_ DC (Figure 1e), although the protocols varied regarding speed and time required to produce the crowns and bur milling path. In total, two partially sintered zirconia discs were used to produce all crowns. Twenty crowns were milled per disc, which were sub-divided into the three milling protocols (6 to 7 crowns for each protocol per disc) (Figure 1d). A new set of burs was used for each disc. The partially sintered zirconia discs were dry milled as observed in Figure 1c. The sample size was calculated using the G*Power 3.1.9.4 software and data from a pilot study considering the following parameters: α = 0.05; power = 0.80; effect size = 0.55, number of groups = 3.

After milling, crowns were carefully removed from the ceramic disc and sintered at 1450 °C for 80 min in an inLab Profire furnace (Dentsply Sirona, Bensheim, Germany), according to the YZ HT Speed protocol indicated by the manufacturer.

The external surface of the sintered milled crowns was qualitatively examined using a stereomicroscope (Zeiss Stemi 2000-C, Carl Zeiss Microscopy GmbH, Göttingen, Germany) and a 3D non-contact profiler (VK-X3000, Keyence, Itasca, IL, USA). For the non-contact profiler, the region of interest (ROI) on the occlusal surface of the crown was chosen after the image registration via navigation, using a 20× lens and autofocus. Following this, the Analyzer software version 2.3.0.151 allowed the leveling of the scan and the creation of a 3D surface profile for each milling protocol.

Before cementation, crowns and abutments were sonically cleaned with isopropyl alcohol for 5 min. The dentin analog abutments were etched using 10% hydrofluoric acid (HF) for 60 s (Condac Porcelana, FGM, Joinville, SC, Brazil) in order to produce micro-retention for adhesive bonding, washed for 30 s, air-dried, and a silane bonding agent (Prosil, FGM, Joinville, SC, Brazil) was applied and allowed to evaporate for 60 s [7,16,29]. An adhesive system (ED Primer A + B, Kuraray, Tokyo, Japan) was applied to the dentin analog surface prior to cementation. No treatment was performed on the zirconia crown internal surface.

Resin-based cement pastes (Panavia F, Kuraray, Tokyo, Japan) were mixed and applied to the internal surface of the crown. The crown was placed over the abutment with finger pressure and assembled to a cementation device, in which a static load of 750 g was applied to the occlusal surface for 5 min (aiming to standardize the cement thickness) [7,29]. The excess cement was removed from the margins and light cured (1200 mW/cm^2^) for 20 s at each surface, including the occlusal surface (Radii-cal, SDI Brasil Ind. e Com. Ltd.a, São Paulo, Brazil). Crowns were stored in distilled water at 37 °C for 48 h before the mechanical tests.

Crowns were subjected to fatigue-simulating chewing in a pneumatic mechanical cycling machine (Biopid, Biocycle, São Carlos, Brazil) with a frequency of 2 Hz for 1.5 × 10^6^ cycles in distilled water at 37 °C [26]. The crowns were placed at a 30° inclination in the equipment, and a cyclic load of 200 N was applied to the lingual cusp by a spherical stainless steel piston (6 mm in diameter). After the chewing simulation, crowns were inspected with transillumination and the use of a stereomicroscope to identify the presence of cracks or fractures.

Crowns that survived the chewing simulation were tested in compression using a universal testing machine (EMIC 23-10, Instron, São José dos Pinhais, Brazil). The load was applied by a spherical stainless steel piston (6 mm in diameter) on the occlusal surface of the crown until fracture (recorded in N), at a cross-head speed of 0.5 mm/min in 37 °C distilled water. A polyester strip was placed between the piston and the crown occlusal surface to achieve a more homogeneous stress distribution.

Fracture load (N) data were analyzed using two-parameter Weibull analysis. One parameter is the characteristic fracture load (L_0_) that corresponds to the fracture load for a 63.2% failure probability. The other parameter is the Weibull modulus (m), which describes the relative spread of fracture load data in the lifetime distribution. The m-value could be used to describe the reliability of a ceramic material, as greater values suggest less data variability and greater precision of the probability of failure estimations [30]. The 95% confidence intervals for the Weibull parameters were calculated using the likelihood ratio method, which is a more accurate method when working with smaller sample sizes. Statistical analysis was performed using a reliability software (Weibull++, Reliasoft).

The fractured surface of the crowns was analyzed using a stereomicroscope (Zeiss Stemi 2000-C, Carl Zeiss Microscopy GmbH, Göttingen, Germany), following fractography principles. Further analysis of representative crowns (2 per group) was performed using a scanning electron microscope (SEM; VEGA3, Tescan Co., Ltd., Brno, Czech Republic). Crowns were cleaned and sputter-coated with gold–palladium (Au-Pd) (Q150r ES Quoron Metalizer, Quorum Technologies Ltd., Laughton, UK) before SEM analysis, which was performed with a secondary electron detector (SE, 20.0 Kv).

## 3. Results

### 3.1. Topography Analysis

**Figure 2:**
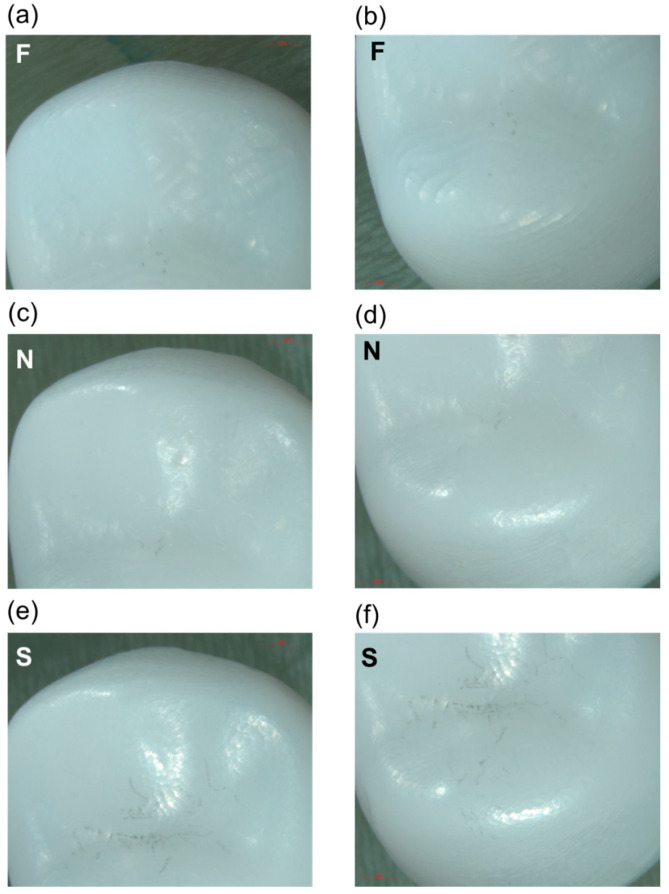
Representative images of the crowns’ occlusal surface for F (**a**,**b**), N (**c**,**d**), and S (**e**,**f**) groups.

Figure 2 shows representative stereomicroscope images of the occlusal surface of the zirconia crowns produced with the different milling protocols. For group F, it is possible to identify prominent marks of the bur path and a rough occlusal anatomy. Groups N and S have a more homogeneous surface and detailed occlusal anatomy. Group S appears slightly smoother than group N.

**Figure 3:**
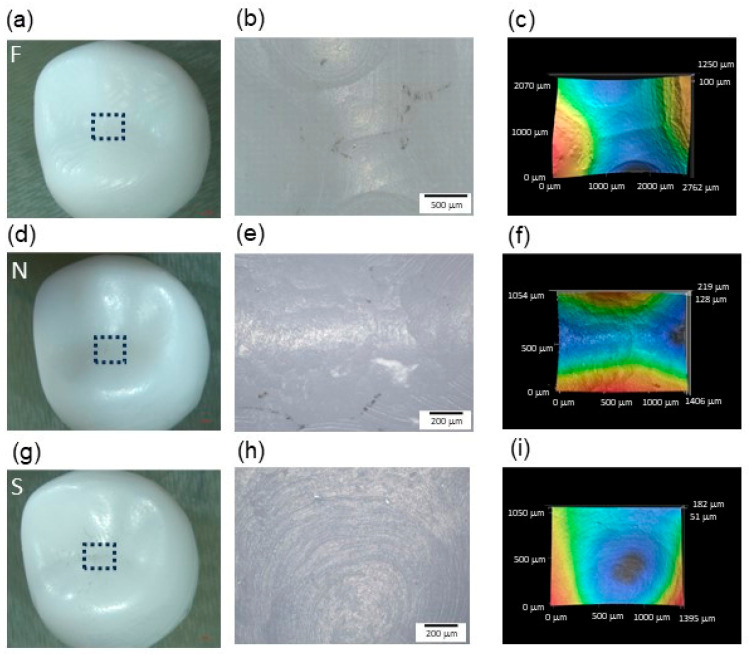
Morphology and surface profile assessed at the central groove of the crowns produced with F (**a**–**c**), N (**d**–**f**), and S (**g**–**i**) milling protocols. The region of interest (ROI—black doted box) on the occlusal surface of the crown was observed using 20× lens. After leveling out the scan images, 3D surface profiles were generated. Images (**b**,**c**), (**e**,**f**), and (**h**,**i**) correspond to the area delimited in the black box of (**a**), (**d**), and (**g**), respectively.

The morphology and surface profile were assessed at the central groove of the crowns, and different bur paths were identified, as observed in Figure 3. Yet it was not possible to associate a milling protocol with only one topography pattern as different bur paths were found for all protocols, varying according to the crown surface and crown analyzed.

### 3.2. Mechanical Analysis

**Figure 4:**
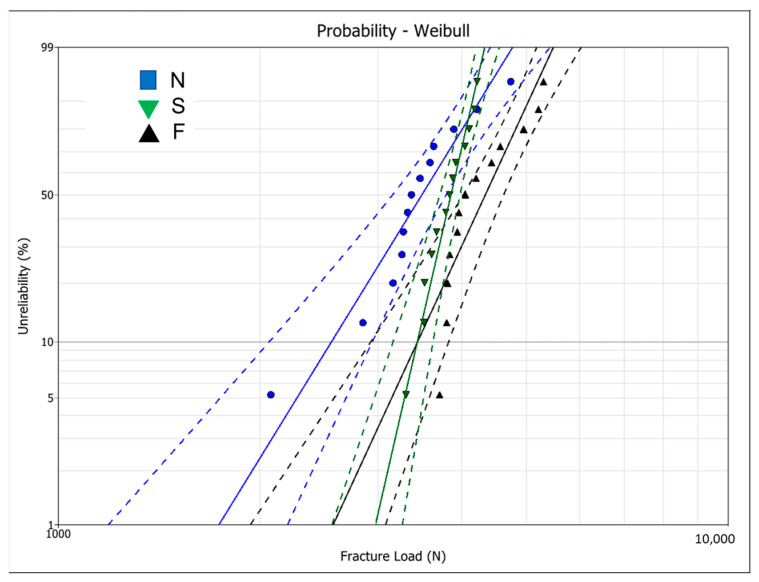
Weibull graph showing the fracture load data for the tested groups. Doted lines correspond to the 95% confidence intervals.

All crowns survived the chewing simulation and were tested under compressive load until fracture. The raw data is available in the Supplementary Materials (Table S1). The Weibull analysis of the fracture load data is presented in Table 3 and Figure 4. Group F had a significantly higher characteristic fracture load (L_0_) than groups N and S as the 95% confidence intervals did not overlap. Group S had the highest Weibull modulus (m), being significantly higher than group N.

**Figure 5:**
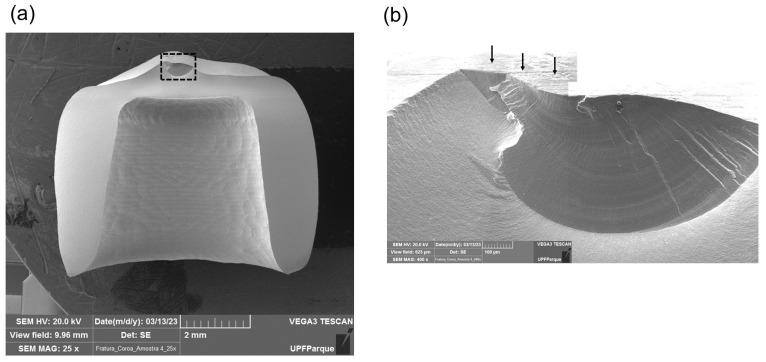
Fractographic analysis of a crown from group N. (**a**) SEM image of the crown showing an area of contact damage in the occlusal surface. (**b**) Closer view of the area delimited by the black box in image (**a**). Black arrows indicate the marks created by the milling burs on the crown occlusal surface.

**Figure 6:**
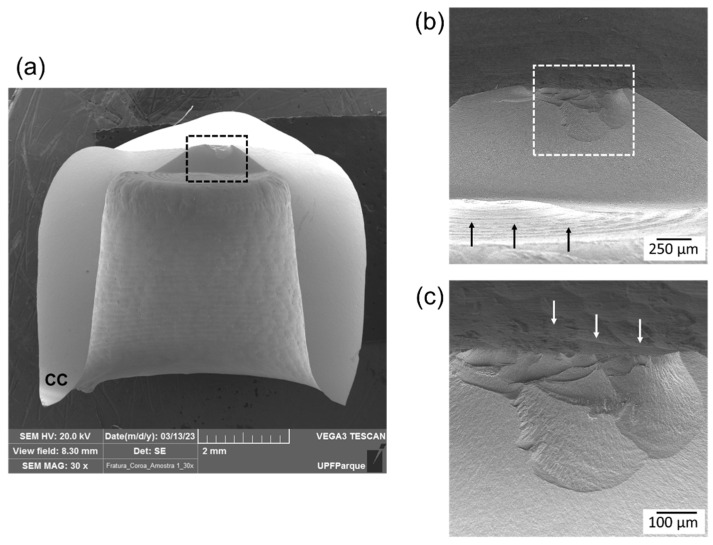
Fractographic analysis of a crown from group S. (**a**) SEM image of the crown showing an area of contact damage in the occlusal surface and the compression curl (CC) located in the cervical margin. (**b**) Closer view of the area delimited by the black box in image (**a**). Black arrows indicate the marks created by the milling burs on the crown intaglio surface. (**c**) Closer view of the fracture origin at the contact damage area delimited by the white box in image (**b**). White arrows point to the multiple origins (cone crack-like features) associated with the marks created by the milling burs on the crown occlusal surface.

**Figure 7:**
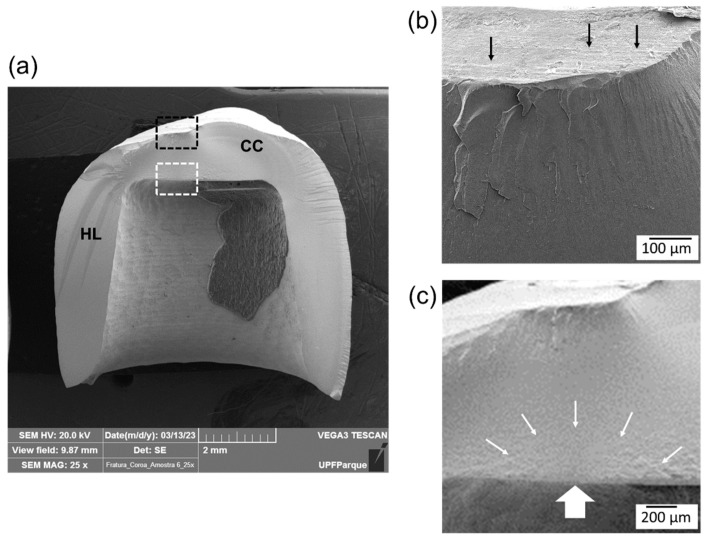
SEM fracture surface images of a crown from group F. (**a**) Fractography suggests the presence of two fracture events. Contact damage was identified in the occlusal surface, as shown in image (**b**). Compression curl (CC) and hackle lines (HL) indicate that the fracture origin was located on the intaglio surface. (**b**) Closer view of the contact damage area delimited by the black box in image (**a**). Black arrows point to the marks created by the milling burs on the crown occlusal surface. (**c**) Closer view of the intaglio surface (white box in image (**a**)) showing the fracture origin, as indicated by the large white arrow and delimited by the small white arrows.

Fractography analysis of the fractured surface of the crowns indicated a similar failure pattern for the three experimental groups. Most frequently, the fracture origin was located on the occlusal surface in the area that was in contact with the loading piston during the mechanical test (Figure 5 and Figure 6), regardless of the protocol. For some specimens, it was also possible to identify a radial crack that originated in the crown intaglio surface combined with occlusal contact damage (Figure 7). The number of fragments observed in group F (53% failed in three fragments) was greater than groups N (27% failed in three fragments) and S (13% failed in three fragments). This suggests that a greater energy was involved in the failure process for group F, as confirmed by its higher fracture load.

## 4. Discussion

Recent improvements in CAD/CAM technology allow for more personalized treatments while also enhancing production efficiency [1]. Nevertheless, it is not clear how the modifications in the CAD/CAM milling process could affect restoration surface conditions and their mechanical behavior. Therefore, the present study qualitatively characterized the topography of monolithic zirconia crowns produced with three different milling protocols and analyzed their failure behavior after being subjected to a chewing simulation. The surface topography, fracture load, and reliability of zirconia crowns were influenced by the milling protocol; hence, the study null hypothesis was rejected.

Zirconia crowns produced with the F protocol had a less homogeneous surface topography than those milled with the N and S protocols. As shown in Figure 2, the F protocol resulted in occlusal grooves and cusps with rough anatomic details. The same sequence of milling burs was used for the three different protocols, which could explain the lack of correlation between the surface pattern created by the burs and the experimental group. Nevertheless, the F protocol was able to produce a monolithic premolar crown in only 12 min, meaning that the contact time of the burs with the ceramic was reduced and less material might be removed. In addition, a faster milling speed could induce a more aggressive process of subtractive material removal that may contribute to surface imperfections [14,17].

Moreover, the greater fracture load values found for crowns produced by fast-milling could be explained by the lack of occlusal anatomy refinement, which may alter the stress distribution and lead to greater ceramic thickness in the central occlusal groove, where the compressive load was subsequently applied. Although the same CAD model was used to produce all monolithic crowns, the CAD/CAM milling process affects the final restoration accuracy, as reported in previous studies [6,10,11]. Therefore, the F protocol may be more suitable for milling dental prosthesis infrastructures that will be subsequently veneered with a glass ceramic or glazed. If monolithic prostheses are being produced, the dentist or lab technician may require additional time for post-milling finishing and polishing procedures [13], which is a disadvantage.

The time required to produce monolithic zirconia crowns with the S protocol (25 min) was greater than the N protocol (18 min), but only minor differences were observed in their surface quality and anatomic refinement, and the fracture load was similar. Nevertheless, the Weibull modulus was greater for crowns milled with the S protocol, which means there is less variability in fracture load data and greater precision in the probability of failure predictions [29,30]. Therefore, when the fracture load for a 5% failure probability was estimated, which is a more clinically relevant parameter, group S had similar values to group F. Milling for a longer time could create a uniform surface with more homogeneous flaw distribution, which thus increased the material reliability.

Besides the differences in the surface topography and mechanical behavior, all monolithic crowns survived the chewing simulation. Crowns were subjected to conditions similar to the ones reported for the posterior areas of the mouth: 200 N load, 2 Hz frequency, and 37 °C humid environment [19,25,26]. The simulation was performed for 1.5 × 10^6^ cycles, which corresponds to approximately 1.5 years of clinical use. Clinical estimation was made considering three chewing episodes per day of 15 min each, resulting in 2700 chewing cycles per day [19,24]. All three milling protocols were able to produce 3Y-PSZ monolithic crown restorations with high survival under the tested conditions, although clinical extrapolations must consider that the methodology applied in the present study did not induce fatigue failure, which is a limitation. 3Y-PSZ has high fracture strength and toughness, which results in high fatigue resistance [3,4,22,23]. Recommendations for future studies include designing fatigue tests with different loading protocols, a greater number of cycles, lateral motion, and artificial saliva. Furthermore, a larger sample size is desired for a more accurate Weibull analysis. Interpretation of the study findings should also consider that crowns were produced using a five-axis production unit for a dental laboratory that has a fully automated tool changer to replace the burs when they lose efficiency, which may lead to a more controlled process.

Considering the high fatigue resistance of monolithic zirconia crowns, compressive load to fracture tests are usually performed to characterize their mechanical behavior [7,25,26,29,31,32]. A study reported fracture load values ranging from 438 N to 3487 N for 3Y-PSZ monolithic crowns of different thicknesses (0.3 mm to 1.5 mm) after mechanical aging [26]. The literature suggested that the ideal thickness of a 3Y-PSZ monolithic prosthesis varies between 0.5 and 1.0 mm [32]. Similar fracture load values have been reported for 3Y-PSZ monolithic crowns in comparison to 4Y-PSZ and 5Y-PSZ cubic-containing zirconias; however, when crowns were previously subjected to a chewing simulation, 3Y-PSZ had the greatest fracture load [31]. Careful fractographic analysis is recommended to validate the findings of laboratory investigations, especially when fracture load tests are applied [24,25,26]. In the present study, a similar failure pattern was observed for the three experimental groups, and it was possible to identify bur marks on the occlusal and intaglio surface of the crowns. Failures originated either from the occlusal contact damage zone (Figure 5 and Figure 6) or, less frequently, from the crown intaglio surface (radial crack) (Figure 7). Both failure modes were previously reported for clinically failed all-ceramic crowns [19,20,21,24].

## 5. Conclusions

The milling protocol affects the topography and fracture behavior of monolithic zirconia crowns. Crowns produced with the fast milling protocol showed a greater fracture load but less homogeneous topography and rough occlusal anatomy. The normal and slow protocols produced crowns with similar fracture load, good surface quality, and anatomic refinement. Crowns milled with the slow protocol had greater reliability.

## Figures and Tables

**Table 1:** Materials investigated in the study.

Material	Brand (Manufacturer)	Composition *	Lot Number
3 mol% yttria partially stabilized zirconia (3Y-PSZ)	VITA YZ HT White (VITA Zahnfabrik, Bad Säckingen, Germany)	ZrO_2_ 90.4–94.5%; Y_2_O_3_ 4–6%; HfO_2_ 1.5–2.5%; Al_2_O_3_ 0–0.3%; Er_2_O_3_ 0–0.5%; Fe_2_O_3_ 0–0.3%	66760
Hydrofluoric acid	Condac Porcelana (FGM, Joinville, SC, Brazil)	10% hydrofluoric acid, water, thickener, surfactant, coloring	040522 040822
Silane coupling agent	Prosil (FGM, Joinville, SC, Brazil)	Ethanolic solution of hydrolyzed 3-Methacryloxypropyltrimethoxysilane	040222
Adhesive system	ED Primer II A + B (Kuraray, Tokyo, Japan)	Liquid A: 2-Hydroxyethyl methacrylate (HEMA), 10-Methacryloyloxydecyl dihydrogen phosphate (MDP), water, N-Methacryloyl-5-aminosalicylic acid (5-NMSA), accelerators liquid B: N-Methacryloyl-5-aminosalicylic acid (5-NMSA), water, catalysts, accelerators	A40061 A5006
Resin cement	Panavia F 2.0 (Kuraray, Tokyo, Japan)	Paste A: MDP, hydrophobic aromatic dimethacrylate, hydrophobic aliphatic dimethacrylate, hydrophilic aliphatic dimethacrylate, silanated silica filler, silanated colloidal silica, dl-Camphorquinone, catalysts, initiators Paste B: Hydrophobic aromatic dimethacrylate, hydrophobic aliphatic dimethacrylate, hydrophilic aliphatic dimethacrylate, silanated barium glass filler, surface-treated sodium fluoride, catalysts, accelerators, pigments	A50258 A60114
Diamond-coated burs	Bur 0.5–1.0–2.5 ZrO_2_ DC (Dentsply Sirona, Bensheim, Germany)		SIR031-18

* Information provided by the manufacturer.

**Table 2:** Experimental groups and description of the CAD/CAM milling protocols.

Group	Protocol	Mode	Time per Crown
S	Slow	Refined	25 min
N	Normal	Conventional	18 min
F	Fast	Fast	12 min

**Table 3:** Characteristic fracture load (L_0_), Weibull modulus (m), fracture load for a 5% failure probability (L_5%_), and respective 95% confidence intervals (95% CI) for the experimental groups.

Group	L_0_ *	L_0_—95% CI	m *	m—95% CI	L_5%_	L_5%_—95% CI
S	4540 a	4194–4889	8.1 ab	5.1–11.7	3286 a	2884–3543
N	3706 b	3337–4091	6.1 b	3.8–8.7	2270 b	1627–2758
F	3943 b	3790–4091	16.3 a	10.1–24.1	3144 ab	2451–3648

* Values followed by the same letters in the same column are statistically similar.

## Data Availability

Data are contained within the article and Supplementary Materials.

## Supplementary Materials

The following supporting information can be downloaded at: https://www.mdpi.com/article/10.3390/ma17122981/s1.
